# Supplementary material for: Methionine supplementing effects on intestine, liver and uterus morphology, and on positivity and expression of Calbindin-D28k and TRPV6 epithelial calcium carriers in laying quail in thermoneutral conditions and under thermal stress
Source: PLoS One. 2021 Jan 15;16(1):e0245615. doi: 10.1371/journal.pone.0245615 (PMC7810298; doi:10.1371/journal.pone.0245615)
Supplement: Attachment — Submitted filename: REBUTTAL LETTER 24_12_2020.pdf [file pone.0245615.s008.pdf]

REBUTTAL LETTER TO THE DECISION LETTER FOR PONE-D-20-01240 24 DECEMBER, 2020

PONE-D-20-19659R1
Methionine supplementing effects on intestine, liver and uterus morphology, and on positivity and expression of Calbindin-D28k and TRPV6 epithelial calcium carriers in laying quail in thermoneutral conditions and under thermal stress
Dr. Ricardo Romão Guerra

Dear Dr. Guerra,

Thank you for submitting your manuscript entitled "Methionine supplementing effects on intestine, liver and uterus morphology, and on positivity and expression of Calbindin-D28k and TRPV6 epithelial calcium carriers in laying quail in thermoneutral conditions and under thermal stress" to PLOS ONE. Your manuscript files have been checked in-house but before we can proceed we need you to address the following issues:

1) Thank you for updating your Data Availability Statement. Can you please clarify whether the main data included in your submission fulfill the minimal data set requirement, or if the data from the real-time PCR analyzes data are necessary for replication?

We define the minimal data set as that which is used to reach the conclusions drawn in the manuscript with related metadata and methods, and any additional data required to replicate the reported study findings in their entirety. This may include:
- The values behind the means, standard deviations and other measures reported
- The values used to build graphs
- The points extracted from images for analysis

If the data from the real-time PCR analyzes data are necessary to meet the Minimal Data Set requirement, please upload the data in a Supporting Information file, or provide the accession codes necessary to access the data from a repository.

Your manuscript has been returned to your account. Please log on to PLOS Editorial Manager at https://www.editorialmanager.com/pone/ to access your manuscript.

Your manuscript can be found in the "Revisions Sent Back to the Author" link under the New Submissions menu. After you have made the changes requested above, please be sure to view and approve the revised PDF after rebuilding the PDF to complete the resubmission process.

Please note that these changes have been requested to comply with submission guidelines and your manuscript will *not* be sent to review until you have fully adhered to our requests. Once your paper has been seen by an Editor we may return it to you for further information or amendments.

We ask that you address this request within 28 days. If you require additional time, please email the journal office. We are happy to grant extensions of up to one month past this due date. If we have not heard from you within 28 days, your manuscript will be withdrawn from Editorial Manager.

Thank you for submitting your work to PLOS ONE.

Kind regards,

Anna Fodor
PLOS ONE

Dear Editor,

In order to fulfill the minimal data set requirement we included real-time PCR analyzes data (slope, efficiency, R2, Threshold, ratio calculation - ΔΔCt method with correction for amplification efficiency and means and standard deviations). The authors consider that with these included data experimente could be replicated. However, if the reviewers consider the need for any additional data, these can be included.

Data:

1) Table 01: Threshold of the real-time PCR reactions.

	Threshold
Beta Actin	0.05819
Calbindin 28	0.08000
TRPV6	0.08998

2) There are attached the Pfaffl tables (06 excel archives) for each tissue (intestine, kidney and uterus) for each genes (Calbindin and TRPV6).

3) Table 2 – Means and Standard Deviations of genic expression found in Figure 5 of article.

Methionine	T°C	calbKidney	SD	calbIntestine	SD	CalbUterus	SD
	20	0,5977	0,2856	6,6536	6,2350	1,2973	0,6958
	24	0,2166	0,1017	7,8677	6,8155	3,3484	2,4808
	28	0,8110	0,6868	3,9541	3,1561	3,5682	3,8387
100,000	32	0,1696	0,0404	8,0661	5,5206	2,4366	2,3557
	20	0,3139	0,2851	1,8712	1,1394	2,3757	3,0204
	24	1,4397	1,3624	4,8613	4,0766	2,2486	2,8014
	28	0,4051	0,1499	3,0245	0,7070	0,9845	0,9837
120,000	32	0,4711	0,3634	2,1525	1,2865	2,0331	0,7065

Methionine	T°C	TRPV6Kidney	SD	TRPV6Intestine	SD	TRPV6Uterus	SD
	20	0,6483	0,3712	0,3797	0,4810	0,5763	0,4287
	24	0,1626	0,0998	0,7702	1,2129	1,3812	1,1002
	28	0,5520	0,3912	0,0214	0,0157	1,1497	1,3400
100,000	32	0,2008	0,0362	0,1672	0,0862	9,1858	12,8536
	20	0,4421	0,3431	0,1230	0,1011	1,9994	2,3097
	24	0,8865	0,5020	0,1371	0,1640	0,3407	0,2132
	28	0,2653	0,1280	0,0203	0,0175	1,0796	0,8247
120,000	32	0,5067	0,1872	0,0517	0,0654	1,4722	1,5425
