# Supplementary material for: Methionine supplementing effects on intestine, liver and uterus morphology, and on positivity and expression of Calbindin-D28k and TRPV6 epithelial calcium carriers in laying quail in thermoneutral conditions and under thermal stress
Source: PLoS One. 2021 Jan 15;16(1):e0245615. doi: 10.1371/journal.pone.0245615 (PMC7810298; doi:10.1371/journal.pone.0245615)
Supplement: Attachment — Submitted filename: carta de refutagem DBCc.docx [file pone.0245615.s007.docx]

**REBUTTAL LETTER TO THE DECISION LETTER FOR PONE-D-20-01240 DATED 5 MAY 2020.**

PONE-D-20-01240
Effects of methionine supplementing on intestine, liver and uterus morphology, and on positivity and expression of calbindin-D28k and TRPV6 calcium carriers in laying quails in thermoneutral conditions and under thermal stress

**5. Review Comments to the Author

Please use the space provided to explain your answers to the questions above. You may also include additional comments for the author, including concerns about dual publication, research ethics, or publication ethics. (Please upload your review as an attachment if it exceeds 20,000 characters)**

**Reviewer #1:** Effects of methionine supplementing on intestine, liver and uterus morphology, and on positivity and expression of calbindin-D28k and TRPV6 calcium carriers in laying quails in thermoneutral conditions and under thermal stress.

An important nutritional issue of the effect of heat stress on calcium metabolism in quail was studied. Quail are an important poultry species in Brazil. The authors evaluated the effect of different temperatures and methionine levels on quail. They evaluated the treatments on histological intestine, liver, kidney, uterine morphologies and immunochemical and PCR analyses of two gene products involved in calcium metabolism.
Quail is the plural of quail.

R: This alteration was made throughout the text of the article.

It appears that there were 42 birds per treatment. How were they housed? In other words what was the experimental unit?

R: In response to this request, the Material and Methods was added with the following texts:

A total of 504 Japanese quail in production stage (second cycle; 21 days each cycle) from the eighth week of life were used, distributed in a completely randomized design in a 3x4 factorial scheme, with three levels of methionine (100%, 110% and 120%) and four temperature ranges (20 24, 28 and 32°C), with 07 replications with 06 birds each (42 animals for treatment), representing thermoneutral (20, 24 and 28°C) and thermal stress ranges (32°C), totaling twelve treatments. Diets were formulated according to NRC (National Research Council) recommendation (Table 1). The quail started the treatments at eight weeks of age and remained for two cycles (42 days), totaling 98 days of experiment.

The major concerns with the quantitative PCR are:
1. that there is a need to determine the efficiency of the primers used for qPCR. A powerful way to determine whether your qPCR assay is optimized is to run serial dilutions of a template and use the results to generate a standard curve. The template used for this purpose can be a target with known concentration (e.g., nanograms of genomic DNA or copies of plasmid DNA) or a sample of unknown quantity (e.g., cDNA). The standard curve is constructed by plotting the log of the starting quantity of template (or the dilution factor, for unknown quantities) against the CT value obtained during amplification of each dilution. The equation of the linear regression line, along with Pearson’s correlation coefficient (r) or the coefficient of determination (R2), can then be used to evaluate whether your qPCR assay is optimized. This issue may have been addressed in the reference Pfaffl MW. Um novo modelo matemático para quantificação relativa em RT-PCR em tempo 641 real. Pesquisa de ácidos nucleicos. 2001;29(9):e45. Unfortunately, for the authors, the reviewer is much less facile in Portuguese than the authors are in English.

R: We agree with the reviewer. We performed efficiency analyses for all primers. Standard curve for each primer was set up using a pool of all experimental cDNA samples in seven 2x dilution from 25 ng to 0.39 ng. The equation of the linear regression line, with the coefficient of determination (R2) was obtained. The slope of the standard curves were between 3.08 and 3.3, R2 value was above 0.99 and qPCR efficiency range between 100 and 110.8%. All data were normalized to a calibrator sample using the ΔΔCt method with correction for amplification efficiency, according Pfaffl (2001). Details were included in the material and methods. As an example, standard curve and curve of efficiency of TRPV6 are demonstrate below:

2. The concern over the use of a single reference for housekeeping gene.
Other than the issue that β-actin is not always stably transcribed under different treatments and growth conditions, there is a concern about the use of a single housekeeping gene. The use of more than one reference or housekeeping gene has been suggested by a recent publication in PLoS ONE. Jacob F, Guertler R, Naim S, Nixdorf S, Fedier A, Hacker NF, et al. (2013) Careful Selection of Reference Genes Is Required for Reliable Performance of RT-qPCR in Human Normal and Cancer Cell Lines. PLoS ONE 8(3): e59180. doi:10.1371/journal.pone.0059180
The authors state “For establishing a set of reference genes for gene normalization we recommend the use of ideally three reference genes selected by at least three stability algorithms.”
The MIQE guidelines (Stephen A. Bustin, Vladimir Benes, Jeremy A. Garson, Jan Hellemans, Jim Huggett, Mikael Kubista, Reinhold Mueller, Tania Nolan, Michael W. Pfaffl, Gregory L. Shipley, Jo Vandesompele, Carl T. Wittwer. The MIQE Guidelines: Minimum Information for Publication of Quantitative Real-Time PCR Experiments, DOI: 10.1373/clinchem.2008.112797 Published March 2009) referenced by Jacob et al., in the 2013 PLoS ONE manuscript included above, state that “Normalization against a single reference gene is not acceptable unless the investigators present clear evidence for the reviewers that confirms its invariant expression under the experimental conditions described. The optimal number and choice of reference genes must be experimentally determined and the method reported.”
It seems reasonable that the RT qPCR experiments, in the current manuscript under consideration for publication in PLOS, be consistent with publications in PLoS ONE that strongly suggest adherence to MIQE guidelines.

R: The MIQE methodology was used.

The endogenous control Beta Actin, which did not vary among treatments are demonstrated below in the tables with the values of CT and CT Corrected by the Efficiency for the different organs (intestine, kidney and uterus) and their respective statistics, where the absence of significance can be observed:

Below are the treatment codes used in the statistical analyzes that follow on the next pages:

S1n1- 20°C – 100% methionine

S1n3- 20°C – 100% methionina

S2n1- 24°C – 100% methionine

S2n3- 24°C – 120% methionine

S3n1- 28°C - 100% methionine

S3n3- 28°C – 120% methionine

S4n1- 32°C – 100% methionine

S4n3- 32°C – 120% methionine

| **Intestine - CT**  s1n1 | s1n3 | s2n1 | | | s2n3 | s3n1 | | s3n3 | | s4n1 | s4n3 | | |
| --- | --- | --- | --- | --- | --- | --- | --- | --- | --- | --- | --- | --- | --- |
| 21,37 | 22,95 | 23,17 | | | 29,8 | 23,34 | | 23,26 | | 25,78 | 20,33 | | |
| 26,14 | 21,29 | 26,89 | | | 22,62 | 21,39 | | 21,07 | | 29,48 | 23,4 | | |
| 22,68 | 26,73 | 28,57 | | | 21,48 | 27,77 | | 20,39 | | 24,66 | 21,58 | | |
| 27,8 | 25,9 | 23,79 | | | 20,52 | 20,2 | | 21,47 | | 25,29 | 22,79 | | |
| Tukey's multiple comparisons test | | | Mean Diff, | 95,00% CI of diff, | | | Significant? | | Summary | Adjusted P Value | |  |  |
| s1n3 vs. s1n1 | | | -0,2800 | -12,50 to 11,94 | | | No | | ns | >0,9999 | | B-A |  |
| s2n1 vs. s1n1 | | | 1,108 | -11,63 to 13,84 | | | No | | ns | 0,9975 | | C-A |  |
| s2n3 vs. s1n1 | | | -0,8925 | -21,87 to 20,08 | | | No | | ns | >0,9999 | | D-A |  |
| s3n1 vs. s1n1 | | | -1,323 | -19,67 to 17,03 | | | No | | ns | 0,9992 | | E-A |  |
| s3n3 vs. s1n1 | | | -2,950 | -14,35 to 8,447 | | | No | | ns | 0,7324 | | F-A |  |
| s4n1 vs. s1n1 | | | 1,805 | -7,721 to 11,33 | | | No | | ns | 0,8954 | | G-A |  |
| s4n3 vs. s1n1 | | | -2,473 | -8,313 to 3,368 | | | No | | ns | 0,3756 | | H-A |  |
| s2n1 vs. s1n3 | | | 1,388 | -8,762 to 11,54 | | | No | | ns | 0,9728 | | C-B |  |
| s2n3 vs. s1n3 | | | -0,6125 | -19,01 to 17,79 | | | No | | ns | >0,9999 | | D-B |  |
| s3n1 vs. s1n3 | | | -1,043 | -10,84 to 8,753 | | | No | | ns | 0,9923 | | E-B |  |
| s3n3 vs. s1n3 | | | -2,670 | -12,80 to 7,464 | | | No | | ns | 0,7206 | | F-B |  |
| s4n1 vs. s1n3 | | | 2,085 | -12,18 to 16,35 | | | No | | ns | 0,9631 | | G-B |  |
| s4n3 vs. s1n3 | | | -2,193 | -11,80 to 7,417 | | | No | | ns | 0,8093 | | H-B |  |
| s2n3 vs. s2n1 | | | -2,000 | -20,71 to 16,71 | | | No | | ns | 0,9921 | | D-C |  |
| s3n1 vs. s2n1 | | | -2,430 | -10,55 to 5,689 | | | No | | ns | 0,6303 | | E-C |  |
| s3n3 vs. s2n1 | | | -4,058 | -15,53 to 7,417 | | | No | | ns | 0,5052 | | F-C |  |
| s4n1 vs. s2n1 | | | 0,6975 | -9,052 to 10,45 | | | No | | ns | 0,9992 | | G-C |  |
| s4n3 vs. s2n1 | | | -3,580 | -11,42 to 4,263 | | | No | | ns | 0,3264 | | H-C |  |
| s3n1 vs. s2n3 | | | -0,4300 | -16,81 to 15,95 | | | No | | ns | >0,9999 | | E-D |  |
| s3n3 vs. s2n3 | | | -2,058 | -12,01 to 7,894 | | | No | | ns | 0,8591 | | F-D |  |
| s4n1 vs. s2n3 | | | 2,698 | -12,09 to 17,49 | | | No | | ns | 0,9089 | | G-D |  |
| s4n3 vs. s2n3 | | | -1,580 | -18,29 to 15,13 | | | No | | ns | 0,9960 | | H-D |  |
| s3n3 vs. s3n1 | | | -1,628 | -13,83 to 10,57 | | | No | | ns | 0,9758 | | F-E |  |
| s4n1 vs. s3n1 | | | 3,128 | -11,76 to 18,01 | | | No | | ns | 0,8516 | | G-E |  |
| s4n3 vs. s3n1 | | | -1,150 | -14,28 to 11,98 | | | No | | ns | 0,9974 | | H-E |  |
| s4n1 vs. s3n3 | | | 4,755 | -3,217 to 12,73 | | | No | | ns | 0,1852 | | G-F |  |
| s4n3 vs. s3n3 | | | 0,4775 | -6,809 to 7,764 | | | No | | ns | 0,9996 | | H-F |  |
| s4n3 vs. s4n1 | | | -4,278 | -9,764 to 1,209 | | | No | | ns | 0,0965 | | H-G |  |

**Kidney – CT**

|  | s1n1 | s1n3 | s2n1 | | | s2n3 | s3n1 | | s3n3 | | s4n1 | s4n3 | | |
| --- | --- | --- | --- | --- | --- | --- | --- | --- | --- | --- | --- | --- | --- | --- |
|  | 27,33 | 23,11 | 25,33 | | | 29,42 | 22,25 | | 24,7 | | 21,48 | 21,79 | | |
|  | 22,68 | 21,76 | 23,67 | | | 23,2 | 27,2 | | 23,03 | | 21,28 | 24,09 | | |
|  | 27,42 | 26,28 | 22,92 | | | 21,57 | 22,12 | | 21,59 | | 21,08 | 22,45 | | |
|  | 26,02 | 22,28 | 21,89 | | | 22,37 | 22,1 | | 22,3 | | 22,78 | 22,28 | | |
| Tukey's multiple comparisons test | | | | Mean Diff, | 95,00% CI of diff, | | | Significant? | | Summary | Adjusted P Value | |  |  |
| s1n3 vs. s1n1 | | | | -2,505 | -7,878 to 2,868 | | | No | | ns | 0,3133 | | B-A |  |
| s2n1 vs. s1n1 | | | | -2,410 | -10,30 to 5,478 | | | No | | ns | 0,6151 | | C-A |  |
| s2n3 vs. s1n1 | | | | -1,723 | -13,20 to 9,751 | | | No | | ns | 0,9585 | | D-A |  |
| s3n1 vs. s1n1 | | | | -2,445 | -17,10 to 12,21 | | | No | | ns | 0,9354 | | E-A |  |
| s3n3 vs. s1n1 | | | | -2,958 | -11,01 to 5,099 | | | No | | ns | 0,4773 | | F-A |  |
| s4n1 vs. s1n1 | | | | -4,208 | -11,45 to 3,035 | | | No | | ns | 0,1966 | | G-A |  |
| s4n3 vs. s1n1 | | | | -3,210 | -13,13 to 6,712 | | | No | | ns | 0,5722 | | H-A |  |
| s2n1 vs. s1n3 | | | | 0,09500 | -7,983 to 8,173 | | | No | | ns | >0,9999 | | C-B |  |
| s2n3 vs. s1n3 | | | | 0,7825 | -13,40 to 14,97 | | | No | | ns | 0,9999 | | D-B |  |
| s3n1 vs. s1n3 | | | | 0,06000 | -12,41 to 12,53 | | | No | | ns | >0,9999 | | E-B |  |
| s3n3 vs. s1n3 | | | | -0,4525 | -9,545 to 8,640 | | | No | | ns | >0,9999 | | F-B |  |
| s4n1 vs. s1n3 | | | | -1,703 | -9,492 to 6,087 | | | No | | ns | 0,8322 | | G-B |  |
| s4n3 vs. s1n3 | | | | -0,7050 | -8,756 to 7,346 | | | No | | ns | 0,9974 | | H-B |  |
| s2n3 vs. s2n1 | | | | 0,6875 | -6,787 to 8,162 | | | No | | ns | 0,9966 | | D-C |  |
| s3n1 vs. s2n1 | | | | -0,03500 | -8,630 to 8,560 | | | No | | ns | >0,9999 | | E-C |  |
| s3n3 vs. s2n1 | | | | -0,5475 | -2,793 to 1,698 | | | No | | ns | 0,7706 | | F-C |  |
| s4n1 vs. s2n1 | | | | -1,798 | -8,002 to 4,407 | | | No | | ns | 0,6541 | | G-C |  |
| s4n3 vs. s2n1 | | | | -0,8000 | -6,661 to 5,061 | | | No | | ns | 0,9730 | | H-C |  |
| s3n1 vs. s2n3 | | | | -0,7225 | -15,37 to 13,92 | | | No | | ns | >0,9999 | | E-D |  |
| s3n3 vs. s2n3 | | | | -1,235 | -8,511 to 6,041 | | | No | | ns | 0,9308 | | F-D |  |
| s4n1 vs. s2n3 | | | | -2,485 | -14,26 to 9,287 | | | No | | ns | 0,8494 | | G-D |  |
| s4n3 vs. s2n3 | | | | -1,488 | -14,38 to 11,41 | | | No | | ns | 0,9882 | | H-D |  |
| s3n3 vs. s3n1 | | | | -0,5125 | -9,115 to 8,090 | | | No | | ns | 0,9998 | | F-E |  |
| s4n1 vs. s3n1 | | | | -1,763 | -10,75 to 7,229 | | | No | | ns | 0,8821 | | G-E |  |
| s4n3 vs. s3n1 | | | | -0,7650 | -5,774 to 4,244 | | | No | | ns | 0,9553 | | H-E |  |
| s4n1 vs. s3n3 | | | | -1,250 | -6,255 to 3,755 | | | No | | ns | 0,7555 | | G-F |  |
| s4n3 vs. s3n3 | | | | -0,2525 | -5,989 to 5,484 | | | No | | ns | >0,9999 | | H-F |  |
| s4n3 vs. s4n1 | | | | 0,9975 | -3,480 to 5,475 | | | No | | ns | 0,8221 | | H-G |  |

**Uterus - CT**

|  | s2n1 | s2n3 | s3n1 | s3n3 | | s4n1 | | s4n3 |  |  |  |  |  |
| --- | --- | --- | --- | --- | --- | --- | --- | --- | --- | --- | --- | --- | --- |
|  | 20,23 | 18,66 | 18,05 | 19,76 | | 30,42 | | 20,01 |  |  |  |  |  |
|  | 17,83 | 19,2 | 20,92 | 30,31 | | 24,45 | | 17,99 |  |  |  |  |  |
|  | 19,72 | 21,67 | 20,67 | 25,44 | | 19,13 | | 20,67 |  |  |  |  |  |
|  | 11,28 | 18,07 | 19,18 | 20,73 | | 22,78 | | 20,04 |  |  |  |  |  |
|  |  |  |  |  | |  | |  |  |  |  |  |  |
| Tukey's multiple comparisons test | | | | | Mean Diff, | | 95,00% CI of diff, | | | Significant? | Summary | Adjusted P Value |  |
| s1n3 vs. s1n1 | | | | | 1,013 | | -7,816 to 9,841 | | | No | ns | 0,9886 | B-A |
| s2n1 vs. s1n1 | | | | | -2,750 | | -14,71 to 9,207 | | | No | ns | 0,8048 | C-A |
| s2n3 vs. s1n1 | | | | | -0,6150 | | -4,678 to 3,448 | | | No | ns | 0,9570 | D-A |
| s3n1 vs. s1n1 | | | | | -0,3100 | | -5,236 to 4,616 | | | No | ns | 0,9997 | E-A |
| s3n3 vs. s1n1 | | | | | 4,045 | | -11,93 to 20,02 | | | No | ns | 0,7467 | F-A |
| s4n1 vs. s1n1 | | | | | 4,180 | | -10,83 to 19,19 | | | No | ns | 0,6825 | G-A |
| s4n3 vs. s1n1 | | | | | -0,3375 | | -2,738 to 2,063 | | | No | ns | 0,9691 | H-A |
| s2n1 vs. s1n3 | | | | | -3,763 | | -13,03 to 5,507 | | | No | ns | 0,4046 | C-B |
| s2n3 vs. s1n3 | | | | | -1,628 | | -12,41 to 9,159 | | | No | ns | 0,9575 | D-B |
| s3n1 vs. s1n3 | | | | | -1,323 | | -12,22 to 9,576 | | | No | ns | 0,9847 | E-B |
| s3n3 vs. s1n3 | | | | | 3,033 | | -14,16 to 20,22 | | | No | ns | 0,9196 | F-B |
| s4n1 vs. s1n3 | | | | | 3,168 | | -5,743 to 12,08 | | | No | ns | 0,5012 | G-B |
| s4n3 vs. s1n3 | | | | | -1,350 | | -12,03 to 9,332 | | | No | ns | 0,9813 | H-B |
| s2n3 vs. s2n1 | | | | | 2,135 | | -8,709 to 12,98 | | | No | ns | 0,8804 | D-C |
| s3n1 vs. s2n1 | | | | | 2,440 | | -10,81 to 15,69 | | | No | ns | 0,9057 | E-C |
| s3n3 vs. s2n1 | | | | | 6,795 | | -10,66 to 24,25 | | | No | ns | 0,4343 | F-C |
| s4n1 vs. s2n1 | | | | | 6,930 | | -10,04 to 23,90 | | | No | ns | 0,4002 | G-C |
| s4n3 vs. s2n1 | | | | | 2,413 | | -10,92 to 15,74 | | | No | ns | 0,9115 | H-C |
| s3n1 vs. s2n3 | | | | | 0,3050 | | -3,812 to 4,422 | | | No | ns | 0,9990 | E-D |
| s3n3 vs. s2n3 | | | | | 4,660 | | -9,228 to 18,55 | | | No | ns | 0,5446 | F-D |
| s4n1 vs. s2n3 | | | | | 4,795 | | -13,50 to 23,09 | | | No | ns | 0,7242 | G-D |
| s4n3 vs. s2n3 | | | | | 0,2775 | | -4,788 to 5,343 | | | No | ns | 0,9999 | H-D |
| s3n3 vs. s3n1 | | | | | 4,355 | | -7,129 to 15,84 | | | No | ns | 0,4533 | F-E |
| s4n1 vs. s3n1 | | | | | 4,490 | | -13,60 to 22,58 | | | No | ns | 0,7593 | G-E |
| s4n3 vs. s3n1 | | | | | -0,02750 | | -6,583 to 6,528 | | | No | ns | >0,9999 | H-E |
| s4n1 vs. s3n3 | | | | | 0,1350 | | -24,90 to 25,17 | | | No | ns | >0,9999 | G-F |
| s4n3 vs. s3n3 | | | | | -4,383 | | -22,29 to 13,53 | | | No | ns | 0,7683 | H-F |
| s4n3 vs. s4n1 | | | | | -4,518 | | -20,51 to 11,48 | | | No | ns | 0,6724 | H-G |

**Intestine - CT Corrected by the Efficiency**

| s1n1 | s1n3 | s2n1 | s2n3 | s3n1 | s3n3 | s4n1 | s4n3 |
| --- | --- | --- | --- | --- | --- | --- | --- |
| 1 | 0,283636619 | 0,237993253 | 0,001202941 | 0,207818708 | 0,221509766 | 0,029688167 | 2,291960314 |
| 0,022279032 | 1,065879815 | 0,012249873 | 0,369027543 | 0,984176386 | 1,270298813 | 0,001552664 | 0,198108661 |
| 0,351785232 | 0,013917078 | 0,003208179 | 0,916012021 | 0,006072129 | 2,184871577 | 0,072526833 | 0,845796484 |
| 0,005928576 | 0,0269787 | 0,145152928 | 1,969699743 | 2,542336193 | 0,92334649 | 0,043883003 | 0,322239501 |

| Tukey's multiple comparisons test | Mean Diff, | 95,00% CI of diff, | Significant? | Summary | Adjusted P Value |  |
| --- | --- | --- | --- | --- | --- | --- |
| s1n3 vs. s1n1 | 0,002605 | -2,365 to 2,370 | No | ns | >0,9999 | B-A |
| s2n1 vs. s1n1 | -0,2453 | -1,498 to 1,008 | No | ns | 0,8826 | C-A |
| s2n3 vs. s1n1 | 0,4690 | -3,327 to 4,265 | No | ns | 0,9833 | D-A |
| s3n1 vs. s1n1 | 0,5901 | -4,092 to 5,272 | No | ns | 0,9816 | E-A |
| s3n3 vs. s1n1 | 0,8050 | -2,705 to 4,315 | No | ns | 0,8064 | F-A |
| s4n1 vs. s1n1 | -0,3081 | -1,756 to 1,139 | No | ns | 0,8454 | G-A |
| s4n3 vs. s1n1 | 0,5695 | -0,9920 to 2,131 | No | ns | 0,4820 | H-A |
| s2n1 vs. s1n3 | -0,2480 | -1,943 to 1,448 | No | ns | 0,9630 | C-B |
| s2n3 vs. s1n3 | 0,4664 | -3,273 to 4,206 | No | ns | 0,9825 | D-B |
| s3n1 vs. s1n3 | 0,5875 | -3,436 to 4,611 | No | ns | 0,9633 | E-B |
| s3n3 vs. s1n3 | 0,8024 | -2,321 to 3,925 | No | ns | 0,7373 | F-B |
| s4n1 vs. s1n3 | -0,3107 | -1,942 to 1,321 | No | ns | 0,8934 | G-B |
| s4n3 vs. s1n3 | 0,5669 | -3,171 to 4,305 | No | ns | 0,9566 | H-B |
| s2n3 vs. s2n1 | 0,7143 | -2,029 to 3,457 | No | ns | 0,7284 | D-C |
| s3n1 vs. s2n1 | 0,8354 | -2,733 to 4,404 | No | ns | 0,7947 | E-C |
| s3n3 vs. s2n1 | 1,050 | -1,827 to 3,928 | No | ns | 0,4814 | F-C |
| s4n1 vs. s2n1 | -0,06274 | -0,4367 to 0,3112 | No | ns | 0,9339 | G-C |
| s4n3 vs. s2n1 | 0,8149 | -1,948 to 3,578 | No | ns | 0,6412 | H-C |
| s3n1 vs. s2n3 | 0,1211 | -2,105 to 2,348 | No | ns | 0,9999 | E-D |
| s3n3 vs. s2n3 | 0,3360 | -2,853 to 3,525 | No | ns | 0,9927 | F-D |
| s4n1 vs. s2n3 | -0,7771 | -3,421 to 1,867 | No | ns | 0,6436 | G-D |
| s4n3 vs. s2n3 | 0,1005 | -4,996 to 5,197 | No | ns | >0,9999 | H-D |
| s3n3 vs. s3n1 | 0,2149 | -4,656 to 5,086 | No | ns | >0,9999 | F-E |
| s4n1 vs. s3n1 | -0,8982 | -4,520 to 2,723 | No | ns | 0,7600 | G-E |
| s4n3 vs. s3n1 | -0,02057 | -5,902 to 5,861 | No | ns | >0,9999 | H-E |
| s4n1 vs. s3n3 | -1,113 | -3,621 to 1,395 | No | ns | 0,3443 | G-F |
| s4n3 vs. s3n3 | -0,2355 | -5,141 to 4,670 | No | ns | >0,9999 | H-F |
| s4n3 vs. s4n1 | 0,8776 | -2,120 to 3,875 | No | ns | 0,6464 | H-G |

**Kidney - CT Corrected by the Efficiency**

| s1n1 | s1n3 | s2n1 | s2n3 | s3n1 | s3n3 | s4n1 | s4n3 |
| --- | --- | --- | --- | --- | --- | --- | --- |
| 1 | 0,283636619 | 0,237993253 | 0,001202941 | 0,207818708 | 0,221509766 | 0,029688167 | 2,291960314 |
| 0,022279032 | 1,065879815 | 0,012249873 | 0,369027543 | 0,984176386 | 1,270298813 | 0,001552664 | 0,198108661 |
| 0,351785232 | 0,013917078 | 0,003208179 | 0,916012021 | 0,006072129 | 2,184871577 | 0,072526833 | 0,845796484 |
| 0,005928576 | 0,0269787 | 0,145152928 | 1,969699743 | 2,542336193 | 0,92334649 | 0,043883003 | 0,322239501 |

| Tukey's multiple comparisons test | Mean Diff, | 95,00% CI of diff, | Significant? | Summary | Adjusted P Value |  |
| --- | --- | --- | --- | --- | --- | --- |
| s1n3 vs. s1n1 | 0,002605 | -2,365 to 2,370 | No | ns | >0,9999 | B-A |
| s2n1 vs. s1n1 | -0,2453 | -1,498 to 1,008 | No | ns | 0,8826 | C-A |
| s2n3 vs. s1n1 | 0,4690 | -3,327 to 4,265 | No | ns | 0,9833 | D-A |
| s3n1 vs. s1n1 | 0,5901 | -4,092 to 5,272 | No | ns | 0,9816 | E-A |
| s3n3 vs. s1n1 | 0,8050 | -2,705 to 4,315 | No | ns | 0,8064 | F-A |
| s4n1 vs. s1n1 | -0,3081 | -1,756 to 1,139 | No | ns | 0,8454 | G-A |
| s4n3 vs. s1n1 | 0,5695 | -0,9920 to 2,131 | No | ns | 0,4820 | H-A |
| s2n1 vs. s1n3 | -0,2480 | -1,943 to 1,448 | No | ns | 0,9630 | C-B |
| s2n3 vs. s1n3 | 0,4664 | -3,273 to 4,206 | No | ns | 0,9825 | D-B |
| s3n1 vs. s1n3 | 0,5875 | -3,436 to 4,611 | No | ns | 0,9633 | E-B |
| s3n3 vs. s1n3 | 0,8024 | -2,321 to 3,925 | No | ns | 0,7373 | F-B |
| s4n1 vs. s1n3 | -0,3107 | -1,942 to 1,321 | No | ns | 0,8934 | G-B |
| s4n3 vs. s1n3 | 0,5669 | -3,171 to 4,305 | No | ns | 0,9566 | H-B |
| s2n3 vs. s2n1 | 0,7143 | -2,029 to 3,457 | No | ns | 0,7284 | D-C |
| s3n1 vs. s2n1 | 0,8354 | -2,733 to 4,404 | No | ns | 0,7947 | E-C |
| s3n3 vs. s2n1 | 1,050 | -1,827 to 3,928 | No | ns | 0,4814 | F-C |
| s4n1 vs. s2n1 | -0,06274 | -0,4367 to 0,3112 | No | ns | 0,9339 | G-C |
| s4n3 vs. s2n1 | 0,8149 | -1,948 to 3,578 | No | ns | 0,6412 | H-C |
| s3n1 vs. s2n3 | 0,1211 | -2,105 to 2,348 | No | ns | 0,9999 | E-D |
| s3n3 vs. s2n3 | 0,3360 | -2,853 to 3,525 | No | ns | 0,9927 | F-D |
| s4n1 vs. s2n3 | -0,7771 | -3,421 to 1,867 | No | ns | 0,6436 | G-D |
| s4n3 vs. s2n3 | 0,1005 | -4,996 to 5,197 | No | ns | >0,9999 | H-D |
| s3n3 vs. s3n1 | 0,2149 | -4,656 to 5,086 | No | ns | >0,9999 | F-E |
| s4n1 vs. s3n1 | -0,8982 | -4,520 to 2,723 | No | ns | 0,7600 | G-E |
| s4n3 vs. s3n1 | -0,02057 | -5,902 to 5,861 | No | ns | >0,9999 | H-E |
| s4n1 vs. s3n3 | -1,113 | -3,621 to 1,395 | No | ns | 0,3443 | G-F |
| s4n3 vs. s3n3 | -0,2355 | -5,141 to 4,670 | No | ns | >0,9999 | H-F |
| s4n3 vs. s4n1 | 0,8776 | -2,120 to 3,875 | No | ns | 0,6464 | H-G |

**Uterus - CT Corrected by the Efficiency**

| s1n1 | s1n3 | s2n1 | s2n3 | s3n1 | s3n3 | s4n1 | s4n3 |
| --- | --- | --- | --- | --- | --- | --- | --- |
| 1 | 29,024 | 4,928 | 0,189 | 57,322 | 8,145 | 106,493 | 82,946 |
| 40,897 | 84,954 | 18,57 | 26,942 | 1,109 | 30,773 | 124,576 | 13,284 |
| 0,928 | 2,31 | 33,683 | 98,854 | 63,753 | 97,031 | 146,508 | 49,001 |
| 2,835 | 56,265 | 76,792 | 52,229 | 58,45 | 55,228 | 37,662 | 46,01 |

| Tukey's multiple comparisons test | Mean Diff, | 95,00% CI of diff, | Significant? | Summary | Adjusted P Value |  |
| --- | --- | --- | --- | --- | --- | --- |
| s1n3 vs. s1n1 | 31,72 | -39,59 to 103,0 | No | ns | 0,3428 | B-A |
| s2n1 vs. s1n1 | 22,08 | -107,1 to 151,2 | No | ns | 0,9288 | C-A |
| s2n3 vs. s1n1 | 33,14 | -126,8 to 193,1 | No | ns | 0,8581 | D-A |
| s3n1 vs. s1n1 | 33,74 | -120,0 to 187,5 | No | ns | 0,8301 | E-A |
| s3n3 vs. s1n1 | 36,38 | -113,1 to 185,8 | No | ns | 0,7715 | F-A |
| s4n1 vs. s1n1 | 92,39 | -52,06 to 236,8 | No | ns | 0,1573 | G-A |
| s4n3 vs. s1n1 | 36,40 | -107,6 to 180,4 | No | ns | 0,7481 | H-A |
| s2n1 vs. s1n3 | -9,645 | -149,9 to 130,6 | No | ns | 0,9994 | C-B |
| s2n3 vs. s1n3 | 1,415 | -208,7 to 211,6 | No | ns | >0,9999 | D-B |
| s3n1 vs. s1n3 | 2,020 | -192,6 to 196,6 | No | ns | >0,9999 | E-B |
| s3n3 vs. s1n3 | 4,656 | -195,4 to 204,7 | No | ns | >0,9999 | F-B |
| s4n1 vs. s1n3 | 60,67 | -153,0 to 274,4 | No | ns | 0,6687 | G-B |
| s4n3 vs. s1n3 | 4,672 | -178,2 to 187,5 | No | ns | >0,9999 | H-B |
| s2n3 vs. s2n1 | 11,06 | -109,5 to 131,7 | No | ns | 0,9966 | D-C |
| s3n1 vs. s2n1 | 11,67 | -98,94 to 122,3 | No | ns | 0,9926 | E-C |
| s3n3 vs. s2n1 | 14,30 | -97,37 to 126,0 | No | ns | 0,9801 | F-C |
| s4n1 vs. s2n1 | 70,32 | -158,5 to 299,1 | No | ns | 0,6108 | G-C |
| s4n3 vs. s2n1 | 14,32 | -131,1 to 159,7 | No | ns | 0,9950 | H-C |
| s3n1 vs. s2n3 | 0,6050 | -129,7 to 130,9 | No | ns | >0,9999 | E-D |
| s3n3 vs. s2n3 | 3,241 | -9,315 to 15,80 | No | ns | 0,7343 | F-D |
| s4n1 vs. s2n3 | 59,26 | -114,7 to 233,2 | No | ns | 0,5334 | G-D |
| s4n3 vs. s2n3 | 3,257 | -173,0 to 179,5 | No | ns | >0,9999 | H-D |
| s3n3 vs. s3n1 | 2,636 | -117,1 to 122,3 | No | ns | >0,9999 | F-E |
| s4n1 vs. s3n1 | 58,65 | -132,4 to 249,7 | No | ns | 0,6116 | G-E |
| s4n3 vs. s3n1 | 2,652 | -58,60 to 63,91 | No | ns | >0,9999 | H-E |
| s4n1 vs. s3n3 | 56,02 | -112,3 to 224,3 | No | ns | 0,5509 | G-F |
| s4n3 vs. s3n3 | 0,01600 | -164,5 to 164,6 | No | ns | >0,9999 | H-F |
| s4n3 vs. s4n1 | -56,00 | -236,5 to 124,5 | No | ns | 0,6035 | H-G |

The articles listed below also used beta actin as an endogenous control for quails and also found no significance:

1. [F. Nicolas Nazar](https://www.ncbi.nlm.nih.gov/pubmed/?term=Nazar%20FN%5BAuthor%5D&cauthor=true&cauthor_uid=25793369), [Bibiana E. Barrios](https://www.ncbi.nlm.nih.gov/pubmed/?term=Barrios%20BE%5BAuthor%5D&cauthor=true&cauthor_uid=25793369), [Pete Kaiser](https://www.ncbi.nlm.nih.gov/pubmed/?term=Kaiser%20P%5BAuthor%5D&cauthor=true&cauthor_uid=25793369), [Raul H. Marin](https://www.ncbi.nlm.nih.gov/pubmed/?term=Marin%20RH%5BAuthor%5D&cauthor=true&cauthor_uid=25793369)  and [Silvia G. Correa](https://www.ncbi.nlm.nih.gov/pubmed/?term=Correa%20SG%5BAuthor%5D&cauthor=true&cauthor_uid=25793369). Immune Neuroendocrine Phenotypes in *Coturnix coturnix*: Do Avian Species Show LEWIS/FISCHER-Like Profiles?. [PLoS One](https://www.ncbi.nlm.nih.gov/pmc/articles/PMC4368694/). 2015; 10(3): e0120712. doi: [10.1371/journal.pone.0120712](https://dx.doi.org/10.1371%2Fjournal.pone.0120712).

2. [Shinji Hiyama](https://www.ncbi.nlm.nih.gov/pubmed/?term=Hiyama%20S%5BAuthor%5D&cauthor=true&cauthor_uid=23000578),[Toshie Sugiyama](https://www.ncbi.nlm.nih.gov/pubmed/?term=Sugiyama%20T%5BAuthor%5D&cauthor=true&cauthor_uid=23000578), [Seiji Kusuhara](https://www.ncbi.nlm.nih.gov/pubmed/?term=Kusuhara%20S%5BAuthor%5D&cauthor=true&cauthor_uid=23000578), [Takashi Uchida](https://www.ncbi.nlm.nih.gov/pubmed/?term=Uchida%20T%5BAuthor%5D&cauthor=true&cauthor_uid=23000578). Evidence for estrogen receptor expression during medullary bone formation and resorption in estrogen-treated male Japanese quails (*Coturnix coturnix japonica*) [J Vet Sci](https://www.ncbi.nlm.nih.gov/pmc/articles/PMC3467396/). 2012 Sep; 13(3): 223–227. doi: [10.4142/jvs.2012.13.3.223](https://dx.doi.org/10.4142%2Fjvs.2012.13.3.223).

We also improved the description of the PCR technique by changing the text as follows:

**Real-Time PCR (qPCR) for TRPV 6 and Calbindin-D28k**

Duodenum and jejunum, kidney and uterus tissues samples were collected from 6 randomly selected animals per treatment. Samples were immediately frozen in liquid nitrogen and then transferred to a -80ºC ultra-freezer until processing. Tissues were homogenized in lysis buffer and RNA extraction was performed using the PureLink™ RNA Mini Kit (Thermo Fisher Scientific) and the ReliaPrep™ RNA Tissue Miniprep System (Promega). Concentration and purity of the RNA samples were determined by the absorbance ratio 260/280 and 260/230 obtained using a spectrophotometer (Colibri Microvolume Spectrometer, Titertek Berthold). RNA integrity was verified on agarose gel. Reverse transcription was performed using the SuperScript™ IV VILO™ Master Mix with ezDNase™ Enzyme (Invitrogen) according to the manufacturers' recommendations.

Expression of selected genes was accessed using a Stratagene Mx3005P qPCR System (Agilent Technologies). Oligonucleotides were obtained from Japanese quail sequences previously published and beta actin was used as endogenous control (Table 2). PCR reactions were performed using Brilliant III Ultra-Fast SYBR® Green QPCR Master Mix with Low ROX (Agilent Technologies). Standard curve for each primer was set up using a pool of all experimental cDNA samples in seven 2x dilution from 25 ng to 0.39 ng. The slope of the standard curves were between 3.08 and 3.3, R^2^ value was above 0.99 and qPCR efficiency range between 100 and 110.8%. Amplification conditions were 3 minutes at 95°C followed by 40 cycles of 15 seconds at 95°C and 20 seconds at 60°C; 1 minute at 95°; 30 seconds at 55º, and finally 30 seconds at 95º.

Melting curve analysis allowed the evaluation of the primer specificity. Samples were run in triplicate for each sample and relative quantification (target gene/endogenous control) determined their expression. Data were normalized to a calibrator sample using the ΔΔCt method [23] with correction for amplification efficiency.

Table 02
Beta actin.
The forward primer corresponds to gamma actin. None of the sequences correspond to Gallus gallus or Cortunix.
The reverse primer corresponds to beta actin from Gallus gallus but not from Cortunix.
With a forward primer specific for one gene sequence and the reverse primer specific for a different gene sequence it is difficult to accept the quantitative data based on differential transcription of beta actin.
The accession number corresponds to Gallus gallus beta actin.
It is suggested that data, an agarose gel, be presented demonstrating a single amplicon under the conditions of the quantitative amplification and that the amplicons derived from the gel be sequenced in both directions.

We also have checked the alignment of the primer sequences in BLAST and, surprisingly, could not recover to the correct sequence. Then, we have tried to align the sequences by specifically choosing the species *Coturnix japonica* *japonica* (taxid: 93934) and we were able to recover the sequences corresponding to the primers. We found that one of the bases of the reverse primer did not match the original sequence, in the meantime that should not be a problem for the correct annealing to the expected sequence, not compromising, therefore, expression analyses. Primer ID reference in the manuscript was changed following the sequence found in GenBank.

**Table 02.** Sequence of primers used for quantitative PCR – real time for quail.

| Genes | qPCR Primers (5'-3') | GenBank Number |
| --- | --- | --- |
| *Calbindin28* | >GACGGCAATGGGTACATGGA  <TCGGGTGTTAAGTCCAAGCC | XM_015855985.1 |
| *TRPV6* | >CCATCATTGCCACCCTCCTT  <AGCAACAATCTGGGCTCTCC | XM_015873874.1 |
| *Beta Actina* | ˃ACCACTGGCATTGTCATGGACTCT  **˂**ACCACTGGCATTGTCATGGACTCT | XM_015872688.2 |

**˃** = forward, **˂** = reverse

**Reviewer #2: General comment:**
This manuscript (MS, PONE-D-20-01240) that included some interesting results presents scientifically and practically important concepts on the effects of dietar methionine supplemention and thermal stress and would of interest to the readers of the PLOS ONE. Indeed, the requirements in terms of scientific quality are fully respected on academic merit and have been presented according to guidelines for authors of the PLOS ONE. Therefore, the MS has novelty for the PLOS ONE. The MS is clear, easy to follow and well related results with discussion. In overall, although the presentation of this MS appears satisfactory and the MS presented in an intelligible fashion and written in standard English, please see revised file (PONE-D-20-01240_revised).
Comments per Section of the Manuscript:
Title: OK
Abstract: Precise
Introduction: OK. Indeed, there is clear rationale for the study in the paper and the paper contribute to generating knowledge. Therefore, the quality of writing is sufficient in terms of providing the reader with information necessary for understanding research presented.
Methodology: Well elaborated.
Results: Well Elaborated
Discussion: The MS describes a technically sound piece of scientific research with data that supports the conclusions. Experiments conducted rigorously, with appropriate controls, replication, and sample sizes. The conclusions drawn appropriately based on the data presented.

R: Unlike reviewer 1, who criticized the sample number, the reviewer 2 quote: “Experiments performed rigorously, with appropriate controls, replication and sample sizes.”

Bibliography/References: Overall write-up and manuscript quality is OK.
Decision: May be considered for further processing as it is.

The Reviewer 2 sent a file with suggested changes. All of them were accepted according to the file that is attached. Except in the title, since it has been changed.

**Reviewer #3:** The manuscript main objective was to show the effects of supplementation of methionine on different tissues of laying quails under heat stress (HS). However, on the introduction the authors missed to explore some key points, such as: discuss the effects of HS on quail production in deep; explore the link between methionine and HS; a paragraph about calbidin-D28k and TRPV6.

R: We changed the content of the introduction, as shown below, to meet the request of reviewer 3:

…

In tropical climates, such as those found in most of Brazil, laying birds suffer a reduction in their zootechnical indexes as well as an increase in mortality as a result of thermal stress by heat, leading to productive and economic losses in production [1]. This fact is mainly observed in laying quail farming, where the first restrictive factor for eggshell formation is calcium, and calcium is negatively influenced by temperature increase [2].

Calcium comes mainly from intestinal absorption and bone and kidney resorption, which is mobilized from the blood to the uterus very quickly [4,5]; two importants ephitelium calcium carries are calbindi-D28k and TRPV6 [4-7]. Nascimento et al. [8] stated that 70% of the production cost is based on food, and for this reason there is a need to develop balanced diets according to the needs of the birds, enabling them to use the diet with maximum efficiency.

For birds subjected to heat stress, it is necessary to supplement the diets with glycogenic amino acids, such as methionine, cystine and others, wich would alleviate the deleterious effects of thermal stress [9]. Under such conditions of thermal stress, physiological and behavioral changes occur in quail, which severely affect feed intake and cause structural changes in the intestinal epithelium, reducing nutrient digestibility and absorption [10].

…

Materials and methods needs to include the number of quails / treatment, what temperatures are thermoneutral, heat or cold stress, and a better description of when the samples were taken, how long the study lasted, how long the treatments lasted, etc

R: In response to this request, the Material and Methods was added with the following texts:

A total of 504 Japanese quail in production stage (second cycle; 21 days each cycle) from the eighth week of life were used, distributed in a completely randomized design in a 3x4 factorial scheme, with three levels of methionine (100%, 110% and 120%) and four temperature ranges (20 24, 28 and 32°C), with 07 replications with 06 birds each (42 animals for treatment), representing thermoneutral (20, 24 and 28°C) and thermal stress ranges (32°C), totaling twelve treatments. Diets were formulated according to NRC (National Research Council) recommendation (Table 1). The quail started the treatments at eight weeks of age and remained for two cycles (42 days), totaling 98 days of experiment.

…

**Histological Processing**

Histological processing was performed at the Histology Laboratory of the Center for Agrarian Sciences of the Federal University of Paraíba. Biological samples of intestine (duodenum and jejunum), liver, uterus and kidney from 8 randomly chosen animals from each treatment (at least 01 animal of each replication) were collected and fixed in Metacarn (60% methanol, 30% chloroform and 10% acetic acid) for 12h and embedded in paraffin in 98° day of experiment. …..

Description of statistical analyses is really basic and it needs a big upgrade. Models were not included, fixed effects, what analysis was done for each sample, etc.

R: In response to this request, it was included in the Histological Processing methodology:

Histomorphometry analyses were performed by a single histologist to avoid misinterpretation. ANOVA and Tukey's post test at 5% significance level were used to evaluate the influence of methionine supplementation on different temperature types. These analyses were performed by using the SAS^®^ University Edition [21].

R: The statistical analysis of the PCR technique has the following description:

Data were subjected to analysis of variance (ANOVA) and, according to the significance of the F test (P ≤ 0.05), the means were compared by the SNK test (Student-Newman-Keuls) at up to 5% probability of error. These analyses were performed by using the SAS^®^ University Edition [21]. Pearson correlation analysis was also performed using the JMP^®^ Pro 13. Data were represented by means ± standard deviation.

The small conclusions in each section of results and discussion are confusing.

R: The discussion was improved, and the conclusion changed, both in the Summary and in the Conclusion item.

Figure 5 is missing units on the Y axis. The quality of the figure is very low. SD is cut from the figure. SD are very high.

R: The Y axis (relative expression) unit has been added, the quality of the figure has been improved, and the standard deviation is shown by the column bar.

Figure 6 is not in English.

R: the names of the organs were actually in Portuguese, but it has already been translated into English.

I highly recommend look for other references in English instead of Portuguese, and to not include (or minimize the inclusion) of references that were not peer-reviewed (such as thesis, dissertation or conference papers).

R: There was the suppression of theses and dissertations from the references.

In the end, the data does not support the conclusion on the use of 120% of methionine supplementation in heat stressed quails.

R: The conclusion has been changed.

This study brings for the first time histomorphological and expression variations (mRNA and protein) of TRPV6 and Calbindin-D28k in organs involved with absorption, reabsorption and calcium deposition in quail; justifies the methionine supplementation (120%) in laying quails for providing thicker eggshells at heat stress and for improving production (%) in thermoneutrality; and justifies the methionine supplementation (120%) in order to partially reverse the deleterious effects of heat stress in the calcium carriers, and in the digestory and reproductive morphology of laying quail.
